# Recent Advances of Hemorrhage Management in Severe Trauma

**DOI:** 10.1155/2014/638956

**Published:** 2014-01-30

**Authors:** Mohamed El Sayad, Hussein Noureddine

**Affiliations:** ^1^Trauma and Orthopaedics, Wishaw General Hospital, Wishaw, UK; ^2^Trauma and Orthopaedics, Ninewells Hospital, Dundee, UK

## Abstract

Trauma is one of the most common causes of mortality worldwide with a substantial percentage of deaths resulting secondary to haemorrhages, which are preventable and treatable when adequately managed. This paper offers a review of the current literature on how to successfully resuscitate patients with major haemorrhage.

## 1. Introduction 

Trauma is the third most common cause of mortality worldwide and is the leading cause of death in the age group ranging between 1 and 44 years [1]. Among those trauma patients, major haemorrhage is responsible for 30 to 40% of mortality, with up to half of them dying prior to their arrival to hospital [1]. Whilst injuries to the central nervous system are the leading cause of mortality, haemorrhagic shock ranks second despite the fact that it could be preventable and reversible. Within hospital settings, it accounts for more than 80% of the mortality in operating theatres and 50% of the overall mortality in the first 24 hours of admission [1]. Major haemorrhage is defined as the loss of 100% of total blood volume within 24 hours, loss of 50% within four hours, or the loss of 150 mL per minute. Given the above, the importance of successful resuscitation of major haemorrhage becomes paramount [2].

## 2. Early Identification 

Early identification of patients with major haemorrhage is a necessity and the diagnosis should be established promptly. Suffering from hypovolemia due to haemorrhage presents with a variety of clinical features. The American College of Surgeons through their ATLS protocols have traditionally provided a classification for haemorrhagic shock that is divided according to the amount of blood loss; see Table 1 [3]. However, there are limitations to this classification. For example, the clinical signs might be masked in young fit individuals with larger physiological reserves, as well as elderly patients on medications such as beta-blockers that manipulate normal physiological responses.

Introduction of new methods of shock assessment has shown to be more sensitive compared to conventional monitoring of vital signs. The shock index is the ratio of heart rate to systolic blood pressure. Birkhahn et al. proved that the shock index was a more useful tool in diagnosing early haemorrhage. They performed an observational study that included 46 healthy blood-donating individuals; each patient had 450 mLs of blood taken over 20 minutes. Their vital signs were measured before donation, immediately after donation while lying down, and after one and five minutes of standing. The results showed a significant increase in the mean shock index, whilst the vital signs remained within the normal limits despite revealing some extent of variation from predonation readings [4].

Cannon et al. performed a retrospective review of 2445 trauma patients to examine the correlation between shock index and mortality rates. Results showed that patients with a shock index ratio of more than 0.9 had a higher mortality rate of 15.9% compared to a rate of 6.9% in those with a normal shock index. Furthermore, patients whose shock indices measured in the emergency department were higher than those measured in the field had a mortality rate of 9.3% compared to only 5.7% in those who had unchanged shock indices [5].

ROPE is another method of assessment that has also been described; it involves the division of pulse rate by pulse pressure (pulse rate/systolic blood pressure-diastolic blood pressure). Ardagh et al. assessed ROPE as a mean to predict the risk of decompensation in patients with compensated haemorrhagic shock, by undergoing a retrospective study on a cohort of 184 trauma victims. Their results showed that a ROPE value of less than 3.0 was predictive of patients remaining stable while a ROPE value of more than 3.0 was predictive of patients that may develop decompensated haemorrhagic shock [6].

Both methods were assessed by Campbell et al. [7] who carried out a prospective observational study of one hundred and sixteen healthy individuals that donated one unit of blood (420 mLs); both their heart rate and blood pressure were measured immediately before and after donating the blood. Their shock index and ROPE measurements were calculated and the mean of both measurements increased significantly by 12.2%. This study showed that the rope and shock indices correlated with blood loss better than conventional observation monitoring and that they are suitable methods to detect early blood loss.

Subsequent to diagnosing hypovolemia due to haemorrhage, it becomes imperative to identify the source of bleeding. This can be achieved by clinical examination (reduced breath sounds, abdominal or pelvic tenderness, deformed limbs, etc.), bedside investigations in the emergency department (trauma series X-rays, fast scan), or radiological investigation most notably whole body CT scan which is very helpful in rapid diagnosis after patient stabilization [8].

## 3. Damage Control Resuscitation (Prevention and Treatment of the Lethal Triad)

Our approach to major haemorrhage mainly stems from experiences accumulated during periods of war. The conflicts in both Afghanistan and Iraq have inspired us to implement changes to current practice. The military has developed a trauma resuscitation protocol of their own similar to the ATLS called Battlefield Advanced Trauma Life Support (BATLS). This is applied to the management of military trauma victims where the starting point is stopping catastrophic haemorrhage, ensued by the conventional ABCDE ATLS algorithm [9].

For appropriate resuscitation to be established, it is important to understand the severe pathophysiological derangements of the exsanguinating patients, in terms of hypothermia, coagulopathy, and acidosis also known as the lethal triad. Bleeding patients with these findings have up to 90% mortality rate requiring massive transfusion [10]. We will discuss each of the elements of the lethal triad in turn.

### 3.1. Hypothermia

Hypothermia secondary to trauma is a common occurrence; it is due to the inability of the body to generate heat, because of altered central thermoregulation, blocked shivering response, and reduction of metabolic activity at the cellular level. Resuscitation is also a common reason for hypothermia. Cold intravenous fluid administration and patient exposure during initial evaluation as well as peritoneal exposure during laparotomies in the operating theatres are all major contributors. Hypothermia is clinically significant when the core temperature is less than 36 degrees for more than 4 hours.

Hypothermia exacerbates coagulopathy by affecting platelet function; the imbalance of thromboxane and prostacyclin reduces the response of platelet activation. Hypothermia also reduces the enzyme activation pathway of the coagulation cascade. At 35°C, factors eleven and twelve would only function at 65% of normal [11]. Trauma victims with a core temperature below 35°C have a poor prognosis, and those with a core temperature of 32°C have a 100% mortality rate [11]. Recent data from the 31st Combat Support Hospital in Iraq showed that 18% of 2848 trauma patients were hypothermic and that hypothermia is significantly correlated with admission GCS, tachycardia, hypotension, low hematocrit, and acidosis thus making it an independent contributor to overall mortality [12].

Treatment of the hypothermic patient could be carried out passively or actively. Passive warming entails preventing further heat loss, by means such as covering the patients and warming the operating or resuscitation room. Active warming involves covering the trauma victims with warming blankets and administering warmed intravenous fluid or blood and warm body cavity lavage, aiming to keep core temperature at 36 degrees.

### 3.2. Acidosis

Metabolic acidosis is due to the prolonged inadequate tissue perfusion. This leads to cellular metabolism converting from aerobic to anaerobic and the increased production of lactate causing a reduction in pH. This reduction in pH has a negative effect on cardiac contractility.

The effect of the acidosis on the coagulation cascade was shown by Meng et al. They measured the activity of recombinant factor VIIa on phospholipids and platelets, and the results showed that it was reduced by 90% when the pH was reduced from 7.4 to 7 [13]. Martini et al. showed the effect of acidosis on coagulation by infusing 12 pigs with 0.2 mol/L of hydrochloric acid until their pH was 7.1. Ringer lactate was continually transfused to maintain that pH. Blood samples were taken at baseline and at 15 minutes after acidosis induction. Coagulation function was assessed by measuring the prothrombin time (PT), partial thromboplastin time (PTT), and thrombin generation. They concluded that acidosis reduced fibrinogen concentration to 66% ± 2%, decreased platelet counts to 49% ± 4%, and decreased thrombin generation to 60% ± 4%. Acidosis also prolonged PT and PTT by about 20% [14].

A sensitive means of assessing tissue oxygenation is by measuring the base deficit, which is a measure of the number of millimoles of base required to correct the pH of one liter of whole blood to 7.4 and its normal value is −3 to +3. Davis et al. demonstrated that measurement of the base deficit was more accurate in deciding the clearance of the acidosis after trauma shock than PH measurement. They performed a retrospective analysis of 674 trauma victims that had both their pH and base deficits measured during their admission to hospital. The base deficit was significantly different between the survivors and the nonsurvivors at all of the time intervals at which it was measured. However, the same differences were not noted when the pH was assessed making it a less reliable predictor of outcome [15].

Treatment of acidosis mainly constitutes restoration of the circulation to maintain tissue perfusion. Lier et al. provided a comprehensive review of the effect of acidosis on coagulation and recommended neutralization of the acidosis to improve the coagulopathy especially when the trauma victim is receiving a massive transfusion. The recommendation was that buffering could be established by using either sodium bicarbonate or trishydroxymethylaminomethane (THAM), with the latter recording superior results. This is due to its effect on coagulation, as it did not inhibit thrombin formation when compared to sodium bicarbonate, but they both had a negative effect on the conversion of fibrinogen to fibrin [16].

### 3.3. Coagulopathy

Hess et al. reviewed the reasons for the development of acute coagulopathy following trauma. They identified 6 initiators: (1) tissue damage which leads to the exposure of endothelium and initiates the coagulation cascade and fibrinolysis; (2) shock, which is dose dependent to the degree of coagulopathy; this mechanism is not clearly understood; (3) haemodilution from the shift of cellular and interstitial fluid that is deficient in clotting factors into the plasma, and the administration of intravenous fluid, which also interrupts clot formation and the transfusion of red blood cells, (4) inflammation which interferes in coagulation as it causes monocytes to adhere to platelets, activation of the thrombomodulin-protein C pathway, and the binding of C4b to protein S [17], finally the last 2 initiators which are (5) hypothermia and (6) acidosis as discussed previously.

Acute coagulopathies are found in one in four trauma patients as shown by Brohi et al. They underwent a retrospective review of 1088 patients that were admitted to their trauma centre between the years of 1993 and 1998. These patients had their coagulation profile measured on admission, including PT, APTT, and thrombin time. The results showed that 24.4% of patients had a coagulopathy and that these patients had a mortality of 46%. This was significantly different to the mortality of 10.9% for those with normal clotting [18].

Currently, there are no accurate methods to assess acute trauma coagulopathy. The traditional investigations of PT, PTT, fibrinogen, and platelet count are of limited value during massive hemorrhage as they do not provide enough information about clot formation. Additionally, the platelet count is usually normal during the initial stage of haemorrhage. The current treatment of coagulopathy is by the early empirical transfusion of blood and clotting factors [19].

### 3.4. Massive Transfusion and Fluid Resuscitation

Massive transfusion is defined as transfusion of more than ten units of packed red cells over 24 hours or four units within one hour. Administering blood should be initiated at the scene of the accident, thus avoiding the rapid administration of iv fluids (filler fluid) that has been traditionally promoted by the ATLS guidelines. This involves giving two liters of crystalloids and continuing with packed red blood cells (PRBCs) and fresh-frozen plasma (FFP) if there was transient or no response, with the aim of achieving normotension.

Duke et al. provided data from a retrospective analysis of 307 patients that were admitted to a level one-trauma centre between January 2007 and May 2011. The inclusion criteria were patients with penetrating torso injuries and a systolic blood pressure less than 90 mmHg who were managed by damage control resuscitation and surgery. 175 patients received standard fluid resuscitation (SFR) which was 150 mLs of crystalloid or more; 132 patients received restricted fluid resuscitation (RFR) which was less than 150 mLs of crystalloid before damage control surgery. The results showed that the first group (SFR) received more preoperative fluid then the (RFR) group (2,275 mLs versus 129 mLs) and that they had a higher intraoperative mortality rate (32% versus 9%) and overall mortality rate (37% versus 21%). The higher mortality rate was attributed to the effect of large volume of fluids in diluting clotting factors and reducing blood viscosity and the increase of blood pressure. RFR was beneficial to these patients as it provided them with permissive hypotension (systolic blood pressure of 90) until damage control surgery was achieved [20].

Serious attempts at challenging the clinical validity of the widely accepted concept of conventional fluid resuscitation first surfaced in the mid-1990s. In 1996, Dries published a mini review of summaries in his paper “hypotensive resuscitation” suggesting that “Limited attempts to restore blood pressure improve cardiac output, tissue perfusion, and survival while attempts to restore normal tension with crystalloid result in increased hemorrhage volume and higher mortality” [21]. However, some of the earlier randomized controlled trials did not concur with those findings. For instance, Dutton et al. [22] underwent a study where a cohort of 110 patients presenting in hemorrhagic shock, more than half of which were victims of penetrating trauma, was randomized to one of two fluid resuscitation protocols: target SBP > 100 mm Hg or target SBP of 70 mm Hg. And fluid therapy was titrated to this endpoint until definitive hemostasis was achieved. His results showed similar survival rates in both groups suggesting that resorting to hypotensive resuscitation did not affect mortality [22]. Nevertheless, since then, more evidence has emerged favoring the concept of hypotensive resuscitation. Morrison et al. performed a randomized controlled study on 90 patients that required a laparotomy or thoracotomy following trauma; they were divided into 2 groups; the first group was resuscitated aiming for a mean arterial blood pressure of 65 mm Hg with standard fluid protocol, while the second group was managed by hypotensive resuscitation to a mean blood pressure of 50 mm Hg. The results showed that the second group had a lower requirement of fluid and blood product transfusion, lower postoperative mortality, and reduced postoperative bleeding. And in those of them who did develop a coagulopathy, it was less severe compared to coagulopathic patients of the first group as evidenced by INR measurements [23].

The ratio of packed RBC (PRBC) : fresh frozen plasma (FFP) : platelets (plt) should be 1 : 1 : 1. Two studies showed that there was an increase in survival rate by increasing the ratio of FFP to RBC [24] and platelets to RBC [25]. This is the current military guidelines in massive transfusion [9].

### 3.5. Antifibrinolytics

Crash 2 was a randomized controlled trial, that was performed in 274 hospitals in 40 different countries and involved 20,211 patients to assess the effect of tranexamic acid on death, vasoocclusive events, and blood transfusion in trauma patients. The patients had to be above the age of 16 years, exposed to trauma, and had to have evidence of bleeding in the form of systolic blood pressure of less than 90 mm Hg or a pulse rate of 110 beats per minute. 10,060 patients received tranexamic acid and 10,067 were randomized to the placebo group. The results yielded that tranexamic acid reduced the mortality rate to 14.5% compared to 16% in the placebo group. The conclusion of this trial was that tranexamic acid reduced the risk of death in trauma patients that were at risk of hemorrhage [26].

This conclusion was also echoed by Morrison et al. in the Military Application of Tranexamic Acid in Trauma Emergency Resuscitation study (MATTER) in 2012. The study was designed as a retrospective observational study that looked at a cohort of 896 patients admitted with combat injuries; the cohort was primarily divided into two groups the first included 293 patients who received TXA, and the second included the rest of the cohort who did not receive TXA. Both groups were further subdivided based on whether patients received massive transfusion (identified as 10 or more units of PRBCs within 24 hours). The outcomes that were measured were mortality at 24 hours, 48 hours, and 30 days, as well as the influence of TXA administration on postoperative coagulopathy and the rate of thromboembolic complications. The results showed an absolute reduction of in-hospital mortality by 6.5% in the TXA group compared to the non-TXA group and an absolute reduction of 49% in the TXA massive transfusion group compared to the massive transfusion non-TXA group. This was not completely cost-free though, as the study established that there was a higher rate of thromboembolic events in the TXA and TXA massive transfusion groups compared to their non-TXA counterparts, but it was noteworthy that there were no increased fatalities due to those events.

## 4. Hemorrhage Control and Damage Control Surgery

Despite taking all the above measures, if hemorrhage control is not achieved these resuscitation efforts would be futile. Hemorrhage control should be achieved in a timely fashion, initially by temporary measures such as direct compression, hemostatic dressings, tourniquets or the application of pelvic binders, and finally by definitive surgical control of the source of bleeding.

Temporary measures like the tourniquet, is well recognized and was first documented in 1674. Bellamy believed that 38% of the patients in the Vietnam War that died from extremity hemorrhage could have been salvaged by appropriate application of a tourniquet [27]. Pelvic binders help stabilize exsanguinating patient with open pelvic fractures by reducing their pelvic volume thus increasing intrapelvic pressure providing a tamponade effect to the catastrophic bleeding that is associated with these injuries. Extreme caution needs to be taken with both of these methods as they are both associated with grave complications due to prolonged application; tourniquets can cause neuropraxia, limb ischemia muscle injury, and compartment syndrome. On the other hand, pelvic binders can lead to skin necrosis.

Damage control surgery is a concept that emerged because the physiological derangements of hemorrhagic shock are more important than definitive surgery. It was described by Feliciano et al. as a 3-stage procedure in which the first is to control the bleeding, the second stage is where the patient is transferred to ITU for correction of the lethal triad and stabilization, and the 3rd stage is for definitive surgical repair [28].

## 5. Conclusion

Whilst massive hemorrhage continues to be a major cause of mortality, it is often reversible, and it can be adequately managed by early identification of these patients and the source of their bleeding, approaching them with (CABC) protocol and prevention and treatment of the lethal triad and damage control surgery to stop the bleeding.

## Figures and Tables

**Table 1:** Classification of haemorrhagic shock (ATLS manual American College of Surgeons).

Class of haemorrhagic shock				
	I	II	III	IV
Blood loss (mL)	Up to 750	750–1500	1500–2000	>2000
Blood loss (% blood volume)	Up to 15	15–30	30–40	>40
Pulse rate (per minute)	<100	100–120	120–140	>140
Blood pressure	Normal	Normal	Decreased	Decreased
Pulse pressure (mm Hg)	Normal or increased	Decreased	Decreased	Decreased
Respiratory rate (per minute)	14–20	20–30	30–40	>35
Urine output (mL/hour)	>30	20–30	5–15	Negligible
Central nervous system/mental status	Slightly anxious	Mildly anxious	Anxious/confused	Confused/lethargic
